# Hydrolyzed milk infant formula effectively protects against milk protein allergy: Independent of whey protein source

**DOI:** 10.1002/fsn3.4480

**Published:** 2024-10-18

**Authors:** Qinggang Xie, Sibo Liu, Dongying Cui, Yang Liu, Xiangxin Wang, Ting Cao, Xiaoxi Xu, Bailiang Li

**Affiliations:** ^1^ College of Food Science Northeast Agricultural University Harbin China; ^2^ Heilongjiang Feihe Dairy Co., Ltd. Beijing China

**Keywords:** allergy, butyrate, goat milk infant formulas, gut microbe, hydrolyzed whey protein, immunity

## Abstract

Milk protein sensitivity is a major challenge in infant feeding, especially for infants who cannot receive adequate breastfeeding. Hydrolyzed milk protein is a mainstream way to address this difficulty. The aim of this study was to assess the effect of differences in whey protein concentrate (WPC) source and the degree of hydrolysis on blocking allergy and to analyze the possible mechanisms by which hydrolyzed infant formula (IF) blocks allergy through colony‐metabolism–immunity response. First, we prepared six groups of goat's milk IF with unhydrolyzed, partially, and extensively hydrolyzed WPC, which come from cow's milk WPC and goat's milk WPC. Subsequently, we evaluated their effects on allergy. The results showed that the hydrolyzed IF improved the allergic characteristics of mice, including low levels of total immunoglobulin E (IgE), specific IgE, histamine, and mucosal mast cell protease‐1 (mMCP‐1). Furthermore, the hydrolyzed IF promoted the immune response of T helper 1 (Th1) and regulatory T (Treg) cells by enhancing the messenger RNA (mRNA) expression of T‐box transcription factor 21 (T‐bet) and forkhead box protein P3 (Foxp3), which in turn suppressed the T helper 2 (Th2) overexpressed immune response in allergy (GATA‐binding protein 3 (GATA‐3) and retinoic‐acid‐receptor‐related orphan receptor gamma t (RORγt) mRNA expression, as well as interleukin 4 (IL‐4) and interleukin 5 (IL‐5) levels). Hydrolyzed IF promoted an increase in beneficial gut microbe *Lactobacillus* and *Alistipes*, which in turn promoted an increase in intestinal butyrate levels. The beneficial bacteria and their metabolized butyrate may have suppressed the abundance of the allergy‐characterizing bacterium *Rikenellaceae*‐RC9‐gut‐group. The final result we obtained was that for both cow's milk WPC and goat's milk WPC, at similar levels of hydrolysis, they did not bring about a significant effect on allergy symptoms. The hydrolyzed IF improved the allergic characteristics of mice, the deeper the degree of hydrolysis of WPC, the more obvious the effect of reducing allergic symptoms in model mice.

## Graphical abstract

The hydrolyzed infant formula (IF) effectively prevented the allergic reaction in the model mice. We demonstrated that for both cow's milk whey protein concentrate (WPC) and goat's milk WPC, at similar levels of hydrolysis, they did not bring about a significant effect on allergy symptoms. The hydrolyzed IF improved the allergic characteristics of mice, the deeper the degree of hydrolysis of WPC, the more obvious the effect of reducing allergic symptoms in model mice.

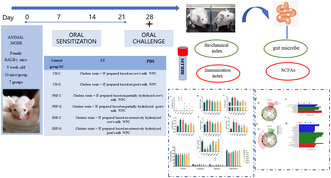

## 1 INTRODUCTION

Food allergy is an adverse immune system‐mediated reaction to the ingestion of a food by the body (Locke et al., 2023). Infant formula (IF) based on cow's or goat's milk is an optional and necessary alternative for infants who cannot be adequately breastfed. However, milk proteins are among the foods that are susceptible to induce allergy. Allergic reactions to milk proteins can lead to a variety of adverse reactions including gastrogut disturbances, rhinitis, wheezing, pulmonary infiltrates, and diarrhea in infants (Golkar et al., 2019; Samady et al., 2018). The global prevalence of cow's milk protein allergy in infants less than 1 year is about 1. 8% to 7. 5%, and it is even higher in developing countries (Mousan & Kamat, 2016). Therefore, the development of hypoallergenic IF is a valuable endeavor in terms of multiple dimensions of effectiveness and cost.

Milk proteins play an antigenic role in inducing allergy due to their two types of epitope structures, linear and conformational epitopes, which can be recognized by the human immune system (Abd El‐Salam & El‐Shibiny, 2021). Destruction of the allergenic structure of milk proteins is the main way to reduce their allergenicity. Therefore, enzymatic hydrolysis of proteins in milk raw materials is an important means to achieve the first dimension, which is to ensure the effectiveness of hypoallergenic IF (Wróblewska et al., 2004). Through enzymatic hydrolysis, the peptide bonds of milk proteins are broken to produce small peptides and amino acids, which reduce the molecular mass of the allergens and thus reduce the allergenicity of milk proteins (Nongonierma & Fitzgerald, 2018; Pushpa et al., 2018). The strength of antigenicity of milk protein hydrolysates has been reported to depend mainly on the type of endonuclease or exonuclease and the degree of hydrolysis. Common endonucleases include trypsin, papain, bromelain, and alkaline protease, while exonucleases are mainly flavored proteases (Kaur et al., 2023). Furthermore, different enzyme combinations and concentrations affect the degree of hydrolysis of milk ingredients (Vorob'ev, 2023).

Goat's milk is increasingly preferred over cow's milk due to its higher digestibility and lower allergenicity. This trend can be observed in the growing number of consumers opting for goat milk‐based infant formula. The preparation of IF needs to be rationalized using whey protein‐related ingredients. The study found comparable levels of whey protein (β‐lactoglobulin) from cow's milk and goat's milk, suggesting that the two may not have an effect on IF allergenicity (Crowther et al., 2019). This is because β‐lactoglobulin is the main component of whey protein that causes sensitization in infants (Zhang, Li, et al., 2023). This suggests that both types of milk whey protein may have a similar impact on the allergenicity of infant formula. The cow's milk and goat's milk whey proteins may not affect the ability of prepared hypoallergenic IF to prevent allergy. Therefore, it is worth studying and discussing whether the addition of either goat's milk or cow's milk WPC to goat's milk has any effect on the allergenicity of IF. Furthermore, it is essential to examine whether the inclusion of hydrolyzed goat's milk WPC or hydrolyzed cow's milk WPC can decrease the allergenicity and determine if there are any discernible differences between them.

A common form of milk protein allergy is a specific immunoglobulin E (IgE)‐mediated immune response (Yu et al., 2016). Milk proteins act as antigens to stimulate the activation of cluster of differentiation 4 + T helper (CD4 + Th) cells, mainly in the T helper 2 (Th2) pathway, which in turn secrete interleukin 4 (IL‐4) and interleukin 13 (IL‐13) cytokines to induce IgE production. IgE binds specifically to mast cell or basophil cell surface receptors to produce histamine and other substances, causing the body to enter a sensitized state (Visser et al., 2010). Subsequently, a large amount of milk proteins bind to the IgE on the cells and undergo a cross‐linking reaction, which in turn triggers an allergic reaction in the host (Bryce, 2016). In recent years, the role of gut microbe in allergy and immunity has been revealed (Fujimura & Lynch, 2015). Significant changes in gut microbe abundance and diversity, and even in microbe composition, have been reported in food‐allergic hosts (Hoskinson et al., 2023). Based on the extensive role of gut microbe and its metabolites in the gut mucosal barrier and immune response, we sought to establish a link between gut microbe and its metabolites and cow's milk protein allergy.

Therefore, the present study first evaluated the effect of goat milk‐based IF prepared from cow's milk WPC and goat's milk WPC in preventing allergy. Subsequently, the effects of hydrolyzed IF on Th1/Th2 and T helper 17 (Th17) cells/Treg homeostasis in mice were analyzed. Furthermore, the gut microbe and metabolite composition of mice were characterized by 16S ribosomal RNA (rRNA) sequencing and gas chromatography–mass spectrometry (GC–MS). Finally, we established a correlation analysis between the gut microbe and metabolites and the immune balance and allergy characteristics after hydrolyzed IF conditioning.

## 2 MATERIALS AND METHODS

### 2.1 Hydrolyzed IF raw materials and composition

The six formulas are composed of 21% whole goat milk powder, 7.5% whey protein concentrate (WPC), 49.05% lactose, 20% mixed vegetable oil, 0.3% complex vitamins, 0.3% complex minerals, 0.2% bicalcium phosphate, 0.5% calcium citrate, 0.2% sodium citrate, and 0.05% potassium chloride. The designed protein content of the six formulas is approximately 11%. The main difference among the six formula groups pertains to the different sources of WPC:

UHF‐C: IF prepared based on unhydrolyzed cow's milk WPC, that the protein content of WPC is 77.18%; UHF‐G: IF prepared based on unhydrolyzed goat's milk WPC, that the protein content of WPC is 79.0%; PHF‐C: IF prepared based on partially hydrolyzed cow's milk WPC, that the protein content of WPC is 77.18% and the degree of hydrolysis is 17.88%, approximately 70.12% of the proteins in WPC have a molecular mass of <6 kD; EHF‐C: IF prepared based on extensively hydrolyzed cow's milk WPC, that the protein content of WPC is 77.18% and the degree of hydrolysis is 47.71%, approximately 90.39% of the proteins in WPC have a molecular mass of <6 kD; PHF‐G: IF prepared based on partially hydrolyzed goat's milk WPC, that the protein content of WPC is 79.0% and the degree of hydrolysis is 18.39%，approximately 71.28% of the proteins in WPC have a molecular mass of <6 kD；EHF‐G: IF prepared based on extensively hydrolyzed goat's milk WPC, that the protein content of WPC is 79.0% and the degree of hydrolysis is 46.10%, approximately 91.30% of the proteins in WPC have a molecular mass of <6 kD.

### 2.2 Animal experiment design

Five‐week‐old female BALB/c mice (*n* = 70) were purchased from Vital River Laboratory Animal Technology Co. Ltd. All mice lived in a controlled environment with four mice per cage with free access to standard laboratory food and water. The experimental environment was set up with a 12‐h light–dark cycle, and temperature and humidity were maintained between 22 ± 2°C and 45 ± 5%. After 1 week of domestication, sensitization assessment of the hydrolyzed IF was carried out. Seventy mice were randomized into seven groups (*n* = 10). On days 0, 7, 14, and 21, mice in the control group (NC group) received 10 μg (micrograms) of cholera toxin (CT). In contrast, the other groups of mice were given the corresponding 20 mg of infant formula (IF) and 10 μg of CT mixed solution 200 μL, respectively. The UHF‐C group received IF based on cow's milk WPC preparation, the UHF‐G group received IF based on goat's milk WPC preparation, the PHF‐C group received partially hydrolyzed IF based on cow's milk WPC preparation, the EHF‐C group received extensively hydrolyzed IF, PHF‐G group received partially hydrolyzed IF based on goat's milk WPC preparation, and EHF‐G group received extensively hydrolyzed IF based on goat's milk WPC preparation. On day 28, control mice were given 0.4 mL of phosphate‐buffered saline (PBS), and the other groups of mice received the corresponding IF to stimulate allergic symptoms. After 30 min, the allergic symptoms of each group were recorded according to the allergy symptom assessment method mentioned in the Pablos‐Tanarro study (Pablos‐Tanarro et al., 2018). This study was approved by the Northeast Agricultural University Animal Care and Welfare Committee under the approved protocol number NEAUEC20230439.

### 2.3 Sample collection

Blood was collected from mice and part of the blood was centrifuged at 1000*g* at 4°C to obtain serum and stored in a −80°C refrigerator. Part of the blood was centrifuged at 2500 × g for 10 min after mixing with heparin, and plasma was collected. Mouse gut contents were collected on sterile tinfoil, placed in sterile freezing tubes, rapidly frozen with liquid nitrogen, and stored at −80°C. The blood was collected by centrifugation at 2500 × *g* for 10 min.

### 2.4 Evaluation of anaphylactic symptoms

According to the method of Liu et al (2020), briefly, grading is based on the following criteria: 0—asymptomatic; 1—scratching and rubbing around the mouth, nose, or ears, slightly moist feces; 2—reduced activity, swelling around the eyes and mouth, shortness of breath, loose and partially formed feces; 3—sedentary activity for more than 1 minute, respiratory distress, severe diarrhea, thin and semiliquid feces; 4—muscle twitching, unresponsive to stimuli, watery and mucusy feces; and 5‐ convulsions or death.

### 2.5 Spleen index

The spleens of each group of mice were collected and weighed and the mice were also weighed. The splenic index was expressed as the ratio of mouse spleen mass to body weight.

### 2.6 Detection of total IgE and specific IgE levels in serum

Total IgE in mouse serum was measured using the IgE Enzyme‐linked Immunosorbent Assay (ELISA) kit (Tiangen, Beijing, China) according to the manufacturer's instructions. Mouse serum‐specific IgE levels were measured using an indirect ELISA according to Zhao's method (2022). Briefly, skimmed milk protein (concentration: 2 μg/100 μL, volume: 100 μL) was added to a 96‐well microtiter plate and incubated at 4°C overnight. After incubation, the plates were washed, blocked with 1.5% gelatin, washed, diluted serum (1:20) was added, incubated overnight at 4°C, and washed three times. One hundred microliters of 3,3′,5,5′‐tetramethylbenzidine (TMB) was added to each well and incubated at 37°C for 30 min in the dark. The color reaction was terminated by adding 50 μL of 4 M sulfuric acid to each well. Absorbance was measured at 450 nm.

### 2.7 Detection of mMCPT‐1 and histamine content

Detection of mouse Mast Cell Protease‐1 (mMCPT‐1) levels was carried out using mMCPT‐1 ELISA kit (Kenuodi). Detection of histamine in mouse plasma was carried out using Histamine ELISA kit (Kenuodi). The results were expressed in terms of concentration.

### 2.8 Histopathological analysis

Histopathological analysis was performed, as described in previous studies (Chen et al., 2021). Ileal and lung tissues were fixed in 4% paraformaldehyde, dehydrated with ethanol, embedded in paraffin wax, and cut into 4‐μm sections, which were subsequently stained with standard hematoxylin–eosin (HE), and the images of the sections were observed using an inverted light microscope (Axio Vert.A1, Carl Zeiss Microscopy GmbH, Germany).

### 2.9 Determination of cytokines in spleen cell culture supernatants

Splenocytes were resuspended in complete medium and cultured in 24‐well microtiter plates (2 × 10^6^ cells/mL). The spleen cells were then stimulated with 4 mg/mL of whey protein, fermented whey protein, and PBS (0.01 M), respectively. The microtiter plates were incubated at 37°C for 72 h in an incubator with 5% carbon dioxide (CO_2_)/95% air. Splenocytes were centrifuged at 250 × *g* at 4°C for 5 min. The supernatants of each group were collected and stored at −80°C until analysis. The contents of IL‐4, IL‐5, interleukin 10 (IL‐10), interleukin 17 (IL‐17), transforming growth factor beta (TGF‐β), and interferon gamma (IFN‐γ) in the supernatants of mouse splenocytes were analyzed by ELISA kit (Tiangen, Beijing, China).

### 2.10 Expression of Th1/Th2 and Th17/Treg‐related transcription factors detected by RT‐qPCR

The expression of Th1/Th2 and Th17/Treg‐related transcription factors in small intestine was detected by reverse transcription quantitative polymerase chain reaction (RT‐qPCR). Total RNA was extracted from 10 mg of mouse small intestine tissue using a tissue total RNA isolation kit (Vazyme Biotech Co. Ltd.). Reverse transcription to complementary DNA (cDNA) was performed, according to the instructions provided in Promega's GoScript™ Reverse Transcription Mix kit. The primers of Foxp3, RORγt, T‐bet, GATA‐3, and glyceraldehyde 3‐phosphate dehyrogenase (GADPH) are shown in Table 1. RT‐qPCR was performed on a QuantStudio^TM^ 3 Real‐Time‐PCR system using Promega's Go Taq®SYBR‐Green qPCR Master Mix. The relative expression of Foxp3, RORγt, T‐bet, and GATA‐3 gene mRNA in each group was analyzed by the 2^−ΔΔ^Ct method, and GAPDH was used as an internal reference gene.

**TABLE 1:** Primer sequences used in reverse‐transcription quantitative PCR assays.

Gene	Primer sequence (5′ → 3′)	
Foxp3	F: TTACTCGCATGTTCGCCTACTTCAG	R: CTCGCTCTCCACTCGCACAAAG
RORγt	F: ACAGCCACTGCATTCCCAGTTT	R: TCTCGGAAGGACTTGCAGACAT
T‐bet	F: ATCACTAAGCAAGGACGGCGAATG	R: ACCAAGACCACATCCACAAACATCC
GATA‐3	F: TCTGGAGGAGGAACGCTAATGGG	R: CGGGTCTGGATGCCTTCTTTCTTC
GADPH	F: GACAGCCGCATCTTCTTGTG	R: AATCCGTTCACACCGACCTT

*Note*: Primer sequences were referenced from Zhao et al (2022).

Abbreviations: F, forward; R, reverse.

### 2.11 Determination of gut microbe

According to the manufacturer's protocol, genomic DNA of the gut microbiome was extracted from 100 mg samples of gut contents using the E.Z.N.A. Stool DNA kit (Omega Bio‐Tek, Norcross, GA, U.S.). Primers 338F and 806R were used to amplify amplified libraries of different regions of the bacterial 16S‐rDNA (ribosomal DNA) gene V3–V4. The primers were designed as forward primer: 5′‐ACTCCTACGG‐GAGGCAGCA‐3′; reverse primer: 5′‐GGACTACHVGGGTATCTAAT‐3′. Library construction was performed using the TruSeq® DNA PCR‐Free Sample Preparation Kit. The libraries were then examined by Qubit and q‐PCR quantification. Sequencing was performed using NovaSeq 6000. The raw data obtained from sequencing were filtered and processed to obtain valid data. Species annotation was performed based on MicroNR libraries and species abundance was obtained at different taxonomic levels.

### 2.12 Determination of short‐chain fatty acids

Gut short‐chain fatty acids (SCFAs) were measured using a Fuli 9720 GC equipped with an Agilent DB‐FFAP capillary column (30 m × 0.25 mm ID × 0.25 μm) along with a flame ionization detector (FID). Accurately weigh 0.8000 ± 0.010 g of cecum contents into a fecal sample box and process it with HALO‐F100 fecal processor to prepare 10% suspension. After thawing, the sample was centrifuged at 8000 rpm (revolutions per minute) at 4°C for 3 min to remove protein and other impurities, and the supernatant was filtered through a 0.22‐μm aqueous filter and then analyzed. The GC conditions were: column temperature 75°C, inlet temperature 250°C, injection volume 1.0 μL, split ratio 5:1; the carrier gas was high‐purity nitrogen at a flow rate of 2.5, and the FID detection temperature was 250°C. The GC conditions were as follows. The SCFAs concentrations were calculated from the peak areas of the samples according to the standard curves obtained for the specific short acid standards.

### 2.13 Statistics analysis

The significance statistics were performed by one‐way analysis of variance (ANOVA), Duncan's multiple comparisons test, and paired‐samples t test of variance with SPSS 22 Version. A *p* value <.05 was considered indicative of statistical significance. All experiments were repeated at least three times, and the data were expressed as mean ± standard deviation (Mean ± SD).

## 3 RESULTS

### 3.1 Hydrolyzed infant formula relieves allergic symptoms in mice independent of WPC from milk source

An allergic mouse model was synergistically induced using cholera toxin (CT) and unhydrolyzed milk proteins to evaluate the protective effect of hydrolyzed IF and unhydrolyzed IF against allergy. We also focused on the differences in allergenicity between whey proteins from cow's milk and goat's milk sources.

**FIGURE 1:**
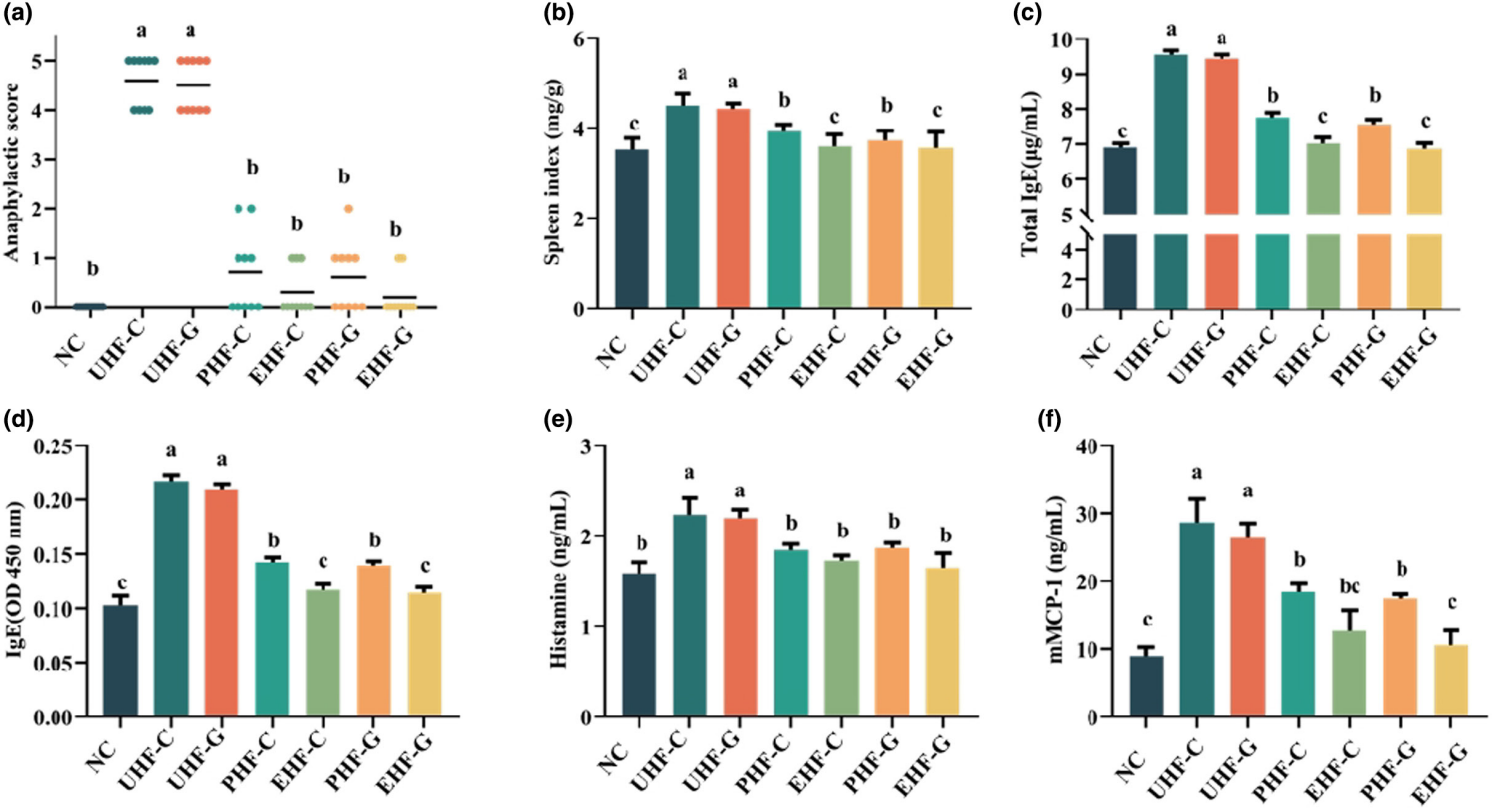
Effectiveness of hydrolyzed IF in alleviating allergic symptoms in mice. (a) Anaphylactic score. (b) Index of spleen. (c) Levels of total IgE in serum. (d) Levels of specific IgE in serum. (e) Levels of histamine in serum. (f) Levels of mMCP‐1 in serum. Data are represented as mean ± SD (a: *N* = 8; b‐f: *N* = 3). Different letters indicate significant difference (*p* < .05). NC: Control group; UHF‐C: IF prepared based on unhydrolyzed cow's milk WPC; UHF‐G: IF prepared based on unhydrolyzed goat's milk WPC; PHF‐C: IF prepared based on partially hydrolyzed cow's milk WPC, EHF‐C: IF prepared based on extensively hydrolyzed cow's milk WPC, PHF‐G: IF prepared based on partially hydrolyzed goat's milk WPC, and EHF‐G: IF prepared based on extensively hydrolyzed goat's milk WPC.

As shown in Figure 1a, mice in the UHF‐C and UHF‐G groups showed typical allergic symptoms such as anaphylactic shock and diarrhea due to the development of anaphylaxis. Compared with the NC group, their allergic scores were significantly increased, indicating successful induction of the allergy model. Whereas, administration of hydrolyzed formulations, both moderate and deep hydrolysis, significantly decreased the allergy scores (Figure 1a). The changes in immune organs showed the same trend as the changes in allergy scores. The unhydrolyzed IF induced a significant increase in spleen weight (Figure 1b), while the hydrolyzed IF did not. However, it was observed that the spleen weight of mice in the EHF‐C and EHF‐G groups was significantly lower than that of mice in the IF with partially hydrolyzed WPC groups (*p* < .05). Meanwhile, there was no significant difference (*p* > .05) between IF with goat's milk WPC and cow's milk WPC.

Subsequently, we investigated the allergy‐related immune responses in mice, mainly including the levels of total IgE, specific IgE, histamine, and mMCP‐1 in mice. The serum levels of total IgE and specific IgE in mice are shown in Figure 1c,d. The levels of total IgE and specific IgE were significantly higher in both the UHF‐C and UHF‐G groups than in the NC group. However, the serum levels of total IgE and milk protein‐specific IgE were significantly lower (*p* < .05) in mice in all hydrolyzed formulation groups, especially in the EHF‐C and EHF‐G groups. Moreover, there was no difference in serum total IgE and specific IgE in both EHF‐C and EHF‐G groups of mice. Histamine and mMCP‐1 are substances expressed on host immune cells when IgE binds to antigenic epitopes, and they are capable of inducing allergic reactions (Hussain et al., 2019). The low levels of IgE in the hydrolyzed IF groups may suggest an inhibition of histamine and mMCP‐1 production. As expected, histamine and mMCP‐1 levels were significantly (*p* < .05) higher in the unhydrolyzed IF groups compared with the NC group, whereas they were significantly lower (*p* < .05) in the hydrolyzed IF groups than in the unhydrolyzed IF groups.

Overall, the hydrolyzed IF significantly reduced IgE levels and decreased the production of allergic inflammatory mediators in mice compared to the unhydrolyzed IF. However, differences between cow's milk and goat's milk ingredients failed to recognize differences in amelioration of allergy. This is because at equal levels of hydrolysis, both goat's and cow's milk WPC had no effect on mouse allergy scores, total IgE, specific IgE, histamine, and mMCP‐1 levels. These results suggest that hydrolyzed IF can alleviate allergic symptoms in mice, but whey protein from cow's milk and goat's milk appears to be irrelevant.

### 3.2 Hydrolyzed IF improves gut organization in mice

**FIGURE 2:**
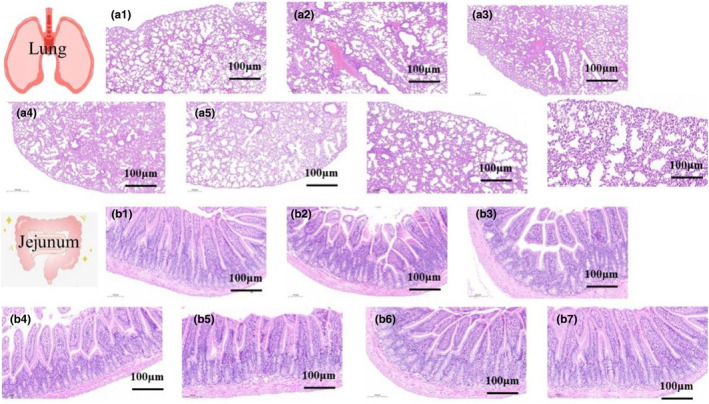
The infant formula (IF) improves lung and ileal tissue changes due to allergies: (a1)–(a7) Histologic analysis of the lungs. (b1)–(b7) Histologic analysis of the lungs. a1 and b1: NC group; a2 and b2: UHF‐C group; a3 and b3: UHF‐G group; a4 and b4: PHF‐C group; a5 and b5: EHF‐C group; a6 and b6: PHF‐G group; a7 and b7: EHF‐G group.

**FIGURE 3:**
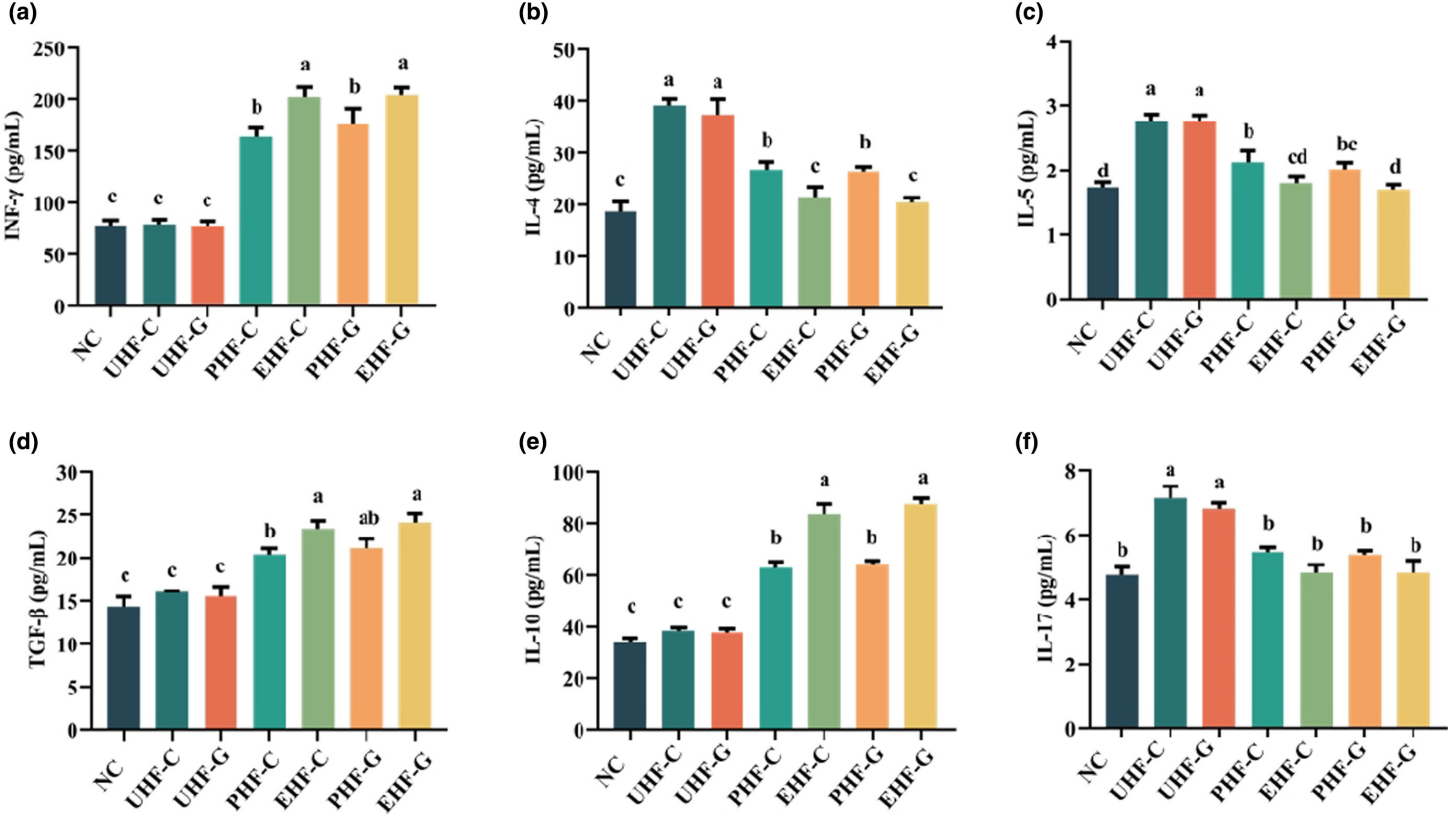
Hydrolyzed IF modulates the level of cytokines for allergic effects in mice. (a) INF‐γ. (b) IL‐4. (c) IL‐5. (d) TGF‐β. (e) IL‐10. (f) IL‐17. Data are represented as mean ± SD (*n* = 3). Different letters indicate significant difference (*p* < .05). NC: Control group; UHF‐C: IF prepared based on unhydrolyzed cow's milk WPC; UHF‐G: IF prepared based on unhydrolyzed goat's milk WPC; PHF‐C: IF prepared based on partially hydrolyzed cow's milk WPC, EHF‐C: IF prepared based on extensively hydrolyzed cow's milk WPC, PHF‐G: IF prepared based on partially hydrolyzed goat's milk WPC, and EHF‐G: IF prepared based on extensively hydrolyzed goat's milk WPC.

**FIGURE 4:**
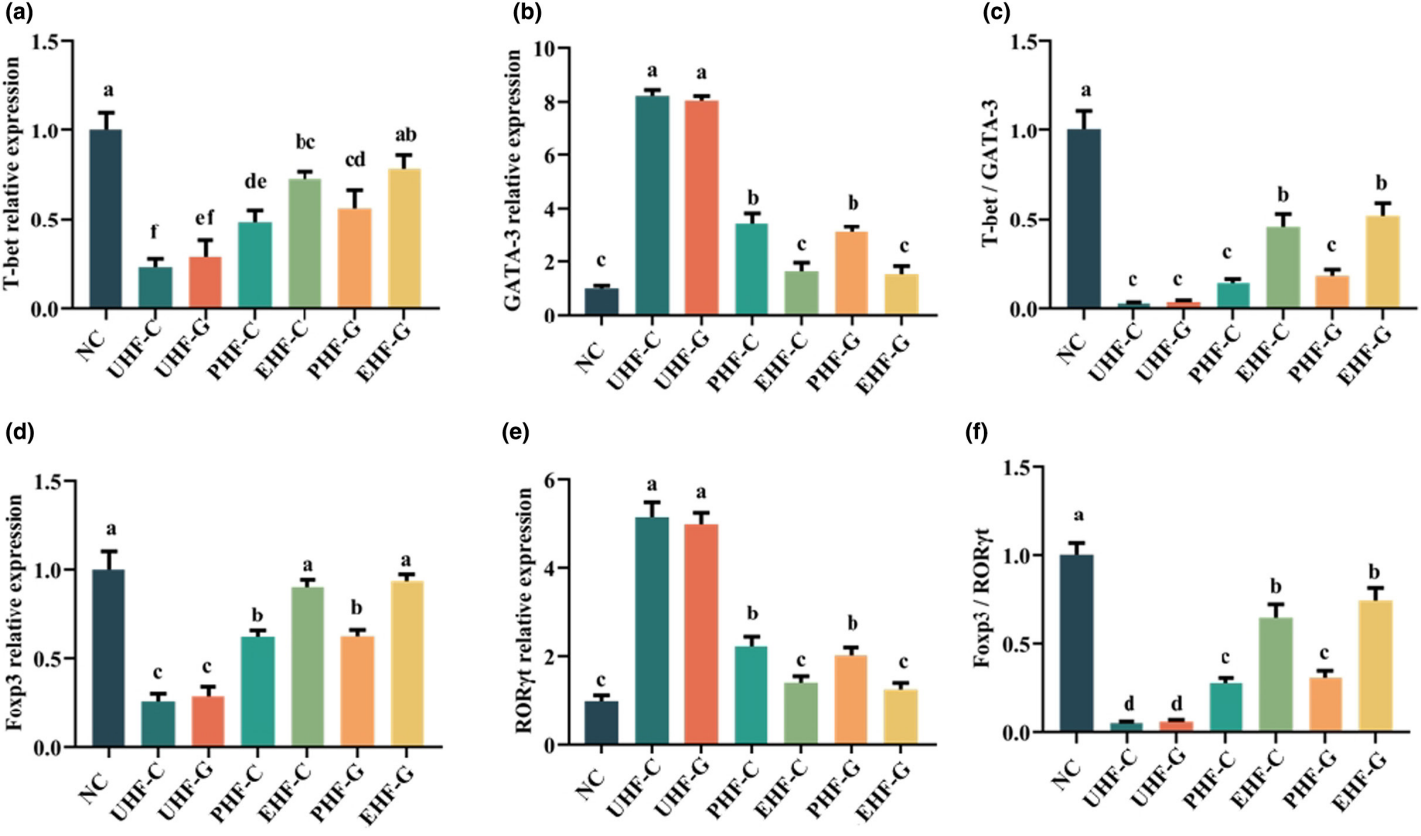
Hydrolyzed IF maintains Th1/Th2 and Th17/Treg homeostasis in mice. (a) T‐bet mRNA expression. (b) GATA‐3 mRNA expression. (c) T‐bet / GATA‐3. (d) Foxp3 mRNA expression. (e) RORγt mRNA expression. (f) Foxp3 / RORγt. Data are represented as mean ± SD (*n* = 3). Different letters indicate significant difference (*p* < .05). NC: Control group; UHF‐C: IF prepared based on unhydrolyzed cow's milk WPC; UHF‐G: IF prepared based on unhydrolyzed goat's milk WPC; PHF‐C: IF prepared based on partially hydrolyzed cow's milk WPC, EHF‐C: IF prepared based on extensively hydrolyzed cow's milk WPC, PHF‐G: IF prepared based on partially hydrolyzed goat's milk WPC, and EHF‐G: IF prepared based on extensively hydrolyzed goat's milk WPC.

Allergic reactions are often accompanied by visible inflammatory reactions in organs or tissues, such as those frequently reported in the lungs and small intestine (Li et al., 2018). Therefore, we observed the histopathological changes in the lungs and ileum of mice in each group using H&E staining. The lung histopathological results indicated that the alveolar structure of mice in the NC group was intact, with no obvious pathological signs. On the contrary, the alveolar structures of mice in the UHF‐C and UHF‐G groups were disrupted and accompanied by inflammatory cell infiltration. We observed that the hydrolyzed IF groups reduced the effects of allergy on lung tissues, but IF with the partially hydrolyzed WPC groups was less effective, especially PHF‐C. Notably, the alveolar structure of mice in the EHF‐C and EHF‐G groups was normal without inflammatory cell infiltration, similar to the NC group. For the histopathological observation of the jejunum, compared with the NC group, the integrity of the villi of mice in the unhydrolyzed IF groups was disrupted, and some inflammatory cell infiltration was present. In contrast, all mice in the hydrolyzed IF groups had intact villi with similar villous structure as the NC group. Overall, allergy induced lesions in the lung and jejunal tissues of mice, including the disruption of alveolar and/or jejunal villi and the presence of inflammatory cell infiltrates. In contrast, administration of the hydrolyzed IF avoided the corresponding damage to some extent (Figures 2, 3, 4).

### 3.3 Hydrolyzed IF regulates levels of immune‐related cytokines

The spleen, as an important immune organ, produces an immune response in allergy‐specific contexts, producing significant changes in cytokine levels. Therefore, we examined the cytokines (IFN‐γ, IL‐4, IL‐5, TGF‐β, IL‐10, and IL‐17) produced by the splenocytes of mice in each group to assess the strength of the immune response. The INF‐γ level was significantly (*p* < .05) higher in the hydrolyzed IF groups than in the unhydrolyzed IF groups and the NC group. Also the EHF‐C and EHF‐G groups were significantly higher (*p* < .05) than the PHF‐C and PHF‐G groups. Previous studies have reported that IFN‐γ inhibits the production of IL‐4 and IL‐5 (Zhao et al., 2022), which is supported by our results. We observed that the levels of IL‐4 and IL‐5 in the hydrolyzed IF groups, which held high levels of IFN‐γ, were significantly lower (*p* < .05) than those in the unhydrolyzed IF groups. In addition, the levels of IFN‐γ, IL‐4, and IL‐5 were lower in the NC group, which may be due to the absence of antigenic uptake, which in turn does not elicit a significant immune response. Subsequently, we investigated the levels of anti‐inflammatory cytokines TGF‐β and IL‐10 derived from T cells, and the levels of TGF‐β and IL‐10 were significantly higher in the hydrolyzed IF groups (*p* < .05).

### 3.4 Hydrolyzed IF improves allergy‐induced Th1/Th2 and Th17/Treg imbalance

Next, we analyzed the expression levels of Th1/Th2 and Th17/Treg‐associated transcription factors in mice, due to the fact that the imbalance of Th1/Th2 and Th17/Treg is the main manifestation in allergic reactions (Tarrant & Finlay, 2023). The expression of T‐bet mRNA was chosen to measure the status of IFN‐γ‐producing Th1 cells, while GATA‐3 was used to indicate IL‐4‐ and IL‐5‐producing Th2 cells. We found significant differences in T‐bet and GATA‐3 mRNA expression between the overall hydrolyzed IF groups and the unhydrolyzed IF groups (*p* < .05). It remains to be noted that there was no statistically significant difference in T‐bet mRNA expression between PHF‐C and UHF‐G groups, although the difference in values between PHF‐C (0.29) and UHF‐G (0.49) was large. Furthermore, the expression of GATA‐3 mRNA in EHF‐C and EHF‐G groups did not differ significantly from that in the NC group (*p* > 0.05). Analysis of the T‐bet and GATA‐3 ratios revealed that the T‐bet/GATA‐3 ratio was significantly lower in all IF groups than in the NC group, but this ratio was also higher in the EHF‐C and EHF‐G groups than in the rest of the IF groups.

In recent years, the balanced relationship between Foxp3 in Treg cells and RORγt in Th17 cells has been shown to play an important role in maintaining gut homeostasis and counteracting allergic reactions (Stephen‐Victor et al., 2020). Thus, we evaluated the mRNA expression of Foxp3 and RORγt in each group of mice. The results showed that the expression of Foxp3 mRNA in the hydrolyzed IF groups was significantly higher (*p* < .05) than in the unhydrolyzed IF groups. In particular, there was no significant difference (*p* > .05) between the EHF‐C, EHF‐G, and NC groups. The expression of RORγt mRNA, in contrast to Foxp3, was significantly lower (*p* < .05) in the hydrolyzed IF groups, whereas it was significantly higher (*p* < .05) in the unhydrolyzed IF groups. The ratio of Foxp3 to RORγt also showed significantly higher (*p* < .05) values in the hydrolyzed IF groups than in the unhydrolyzed IF groups.

Overall, the unhydrolyzed IF was able to disrupt Th1/Th2 and Th17/Treg in mice compared to the unsensitized NC group, while the hydrolyzed IF contributed to the homeostasis between Th1/Th2 and Th17/Treg, especially Th17/Treg. Meanwhile, the effect of the EHF‐C and EHF‐G groups was better than that of the PHF‐C and PHF‐G groups, but there was no difference between whey protein from cow's milk and goat's milk.

### 3.5 Hydrolyzed IF regulates gut microbe

**FIGURE 5:**
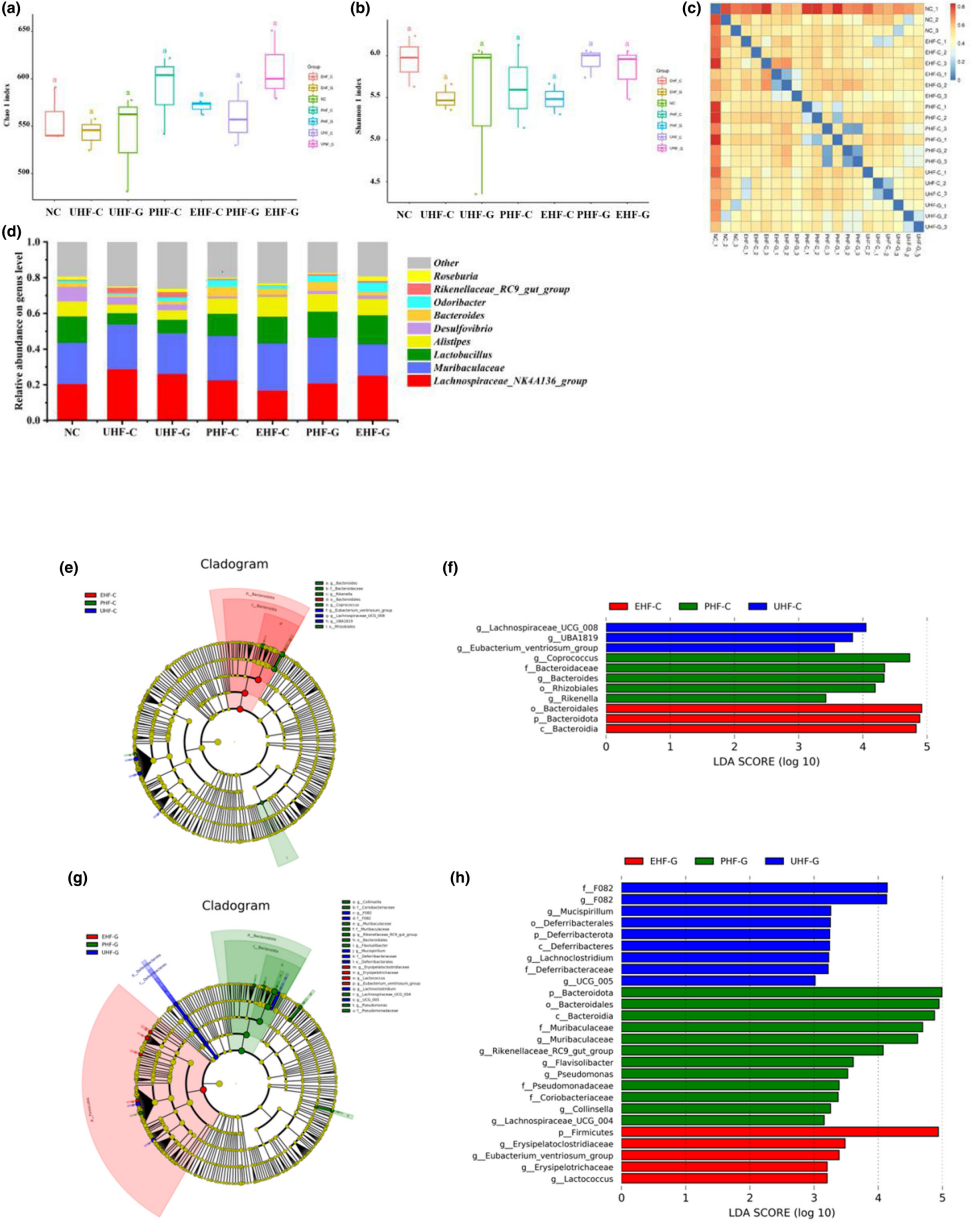
Hydrolyzed IF regulates gut microbe in mice. (a) Chao 1 index. (b) Shannon index. (c) Bray–Curtis. (d) Relative abundance on genus level. The cladogram maps (e) and LDA score maps (f) from LEfSe analysis of UHF‐C, PHF‐C, and EHF‐C groups. The cladogram maps (g) and LDA score maps (h) from LEfSe analysis of UHF‐C, PHF‐C, and EHF‐C groups. Different letters indicate significant difference (*p* < .05). NC: Control group; UHF‐C: IF prepared based on unhydrolyzed cow's milk WPC; UHF‐G: IF prepared based on unhydrolyzed goat's milk WPC; PHF‐C: IF prepared based on partially hydrolyzed cow's milk WPC, EHF‐C: IF prepared based on extensively hydrolyzed cow's milk WPC, PHF‐G: IF prepared based on partially hydrolyzed goat's milk WPC, and EHF‐G: IF prepared based on extensively hydrolyzed goat's milk WPC.

We analyzed the composition of the microbe in the gut contents of each group of mice using 16S rRNA sequencing to assess the effect of allergy on the microbe and to verify that the hydrolyzed IF protects against the damaging effects of allergy on the microbe. Chao 1 index and Shannon index, as an indication of α‐diversity of gut microbe, measured gut microbiota richness and diversity. Figure 5a,b show that the Chao 1 index and Shannon index were not significantly different between groups of mice (*p* > 0.05). It is implied that allergy may cause changes in the abundance of some microorganisms rather than complete removal of certain microorganisms. The β‐diversity analysis, which was chosen to represent the weighted unifrac, revealed good intrapopulation cohesion and significant interpopulation dispersion among the samples (Figure 5c). The weighted unifrac is a means of analyzing differences between groups of microbial communities, taking into account species abundance (Zhang et al., 2017).

Subsequently, we analyzed the composition of each group of samples at the genus level (Figure 5d). All groups of mice contained significant amounts of *Lachnospiraceae*‐NK4A136‐group and *Muribaculaceae*, with both accounting for 42.59%–53.77% of the overall microbe abundance. However, the abundance of *Lachnospiraceae*‐NK4A136‐group and *Muribaculaceae* was similar among the groups and did not differ significantly. Furthermore, *Lactobacillus* and *Alistipes* also had sizable abundance in all groups of mice. They are important and generally probiotic microbiota in the host gut (Ma et al., 2023; Zhang, Liu, et al., 2023). Notably, our results revealed a significant reduction in the abundance of *Lactobacillus* and *Alistipes* in unhydrolyzed IF‐induced allergic mice. The abundance of *Lactobacillus* in UHF‐C and UHF‐G groups was only 42.69% and 50.79 of that in the NC group. While the abundance of *Alistipes* in UHF‐C (55.58%/NC) and UHF‐G groups (63.54%/NC) had similar results. Whereas, in the hydrolyzed IF group, the abundance of *Lactobacillus* and *Alistipes* was significantly higher than that of the unhydrolyzed IF groups and comparable to that of the NC group. In addition, some low‐abundance microorganisms also showed significant differences between the unhydrolyzed and hydrolyzed IF groups, such as *Rikenellaceae*‐RC9‐gut‐group and *Roseburia*. In the NC group, the abundance of *Rikenellaceae*‐RC9‐gut‐group was 0.53%, while it was 3.04% and 3.02% in the UHF‐C and UHF‐G groups, respectively. And *Roseburia* had higher levels mainly in the NC and EHF‐G groups.

The linear discriminant analysis effect size (LEfSe) based on linear discriminant analysis (LDA) was used to estimate the magnitude of the effect of abundance of each species on the differential effect and to screen for differential species between groups (Quince et al., 2017). We subjected UHF‐C, PHF‐C, and EHF‐C to analysis to assess differential species at different levels of hydrolysis for formulation powders prepared with bovine WPC (Figure 5e,f). Differences in formulation powders prepared with goat WPC were obtained by co‐analysis with UHF‐G, PHF‐G, and EHF‐G groups (Figure 5g,h). *Coprococcus*, *Bacteroides*, and *Rikenella* were significantly enriched in the PHF‐G group and *Bacteroidia* were also enriched in the EHF‐G group. In addition, we noted that *Rikenellaceae*‐RC9‐gut‐group was significantly enriched in the PHF‐G group and *Lactococcus* was significantly enriched in the EHF‐G group. However, *Mucispirrillum* and *Lachnoclostridium* were predominantly enriched in the UHF‐G group, and *Lachnospiraceae*‐UCG‐008 and *Eubacterium*‐*ventriosum*‐group in the UHF‐C group.

### 3.6 Altered levels of gut microbe‐associated metabolites–SCFAs

Short‐chain fatty acids (SCFAs) are the main products of fermentation of food components by gut microbe and are critical bacterial metabolites for maintaining host gut health and ameliorating diseases (Koh et al., 2016). Some studies have reported that SCFAs maintain mesenteric lymph node insulin‐like growth factor 1 (IGF‐1) levels, which inhibit Th2 cell differentiation, thereby alleviating dysbiosis‐induced food allergy (Yuan et al., 2021). Based on the observed difference in mouse microbe, we hypothesized that the difference also affected the production of SCFAs. Subsequently, we determined the concentrations of acetate, propionate, and butyrate in the intestines of each group of mice. The results showed that there was a significant difference (*p* < .05) in the butyrate content in the intestines of mice in each group, whereas there was no significant difference (*p* > .05) in the acetate, propionate, and total SCFAs contents. For the butyrate content, the unhydrolyzed IF groups were significantly lower (*p* < .05) than the NC group, suggesting that the allergic reaction reduced the gut butyrate production in mice. Butyrate content in the hydrolyzed groups was significantly higher (*p* < .05) than that in the unhydrolyzed groups, and there was no significant difference between the EHF‐C, EHF‐G, and NC groups (*p* > .05). Furthermore, there was no significant difference (*p* > .05) between different milk ingredients with the same degree of hydrolysis. This may imply that the difference in butyrate content was due to the effect of allergic symptoms and not related to the food ingredients in this study.

### 3.7 Correlation analysis of gut microbe–SCFAs‐allergy characteristics

**FIGURE 6:**
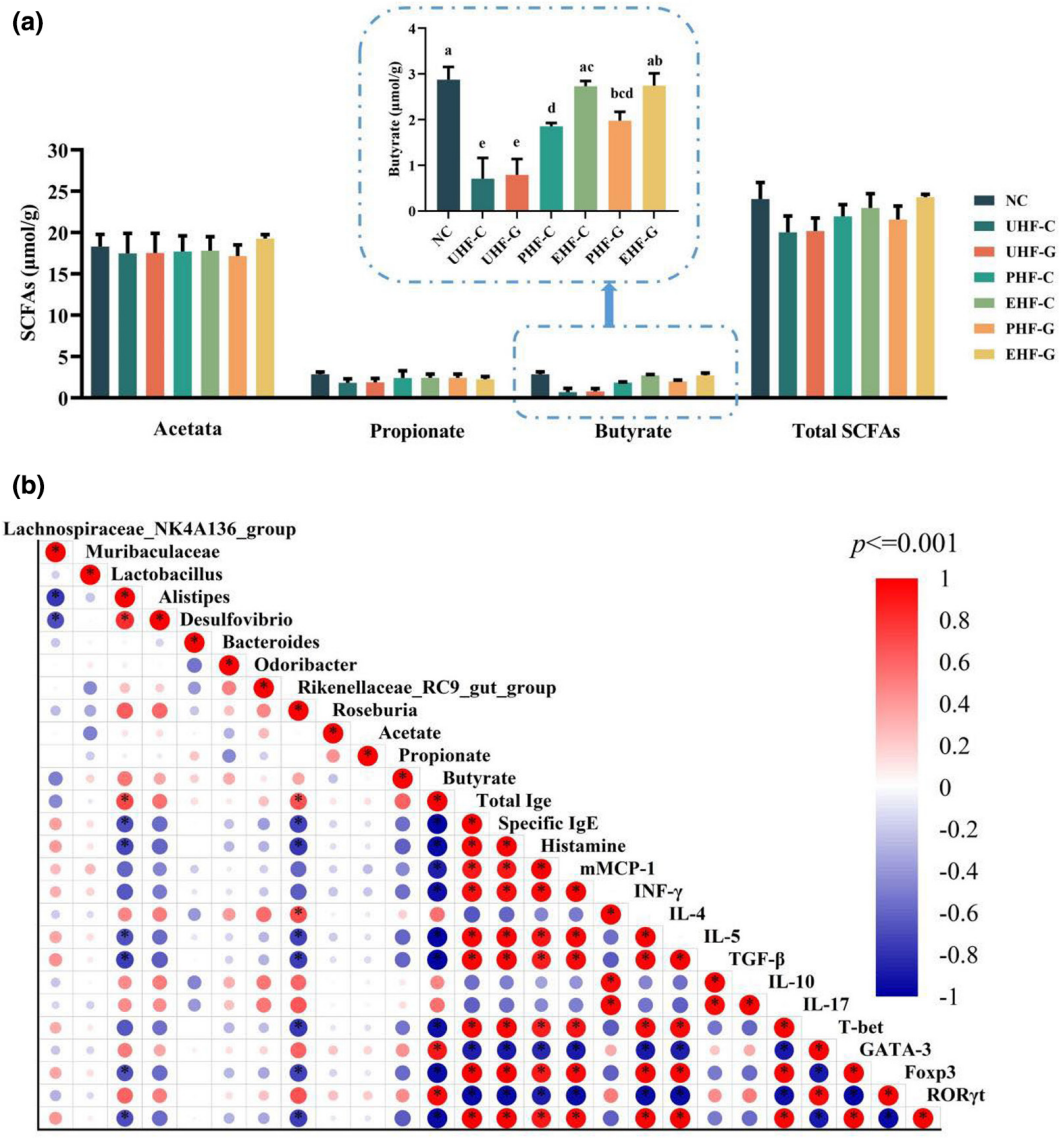
The hydrolyzed IF was able to avoid the allergy‐induced decrease in butyrate concentration. Concentrations of acetate, propionate, and butyrate were examined in all samples and total SCFAs were calculated as the sum of the three. Data are represented as mean ± SD (*n* = 3). Different letters indicate significant difference (*p* < .05). Acetate, propionate, and total SCFAs were not observed to be statistically significant in all samples and are therefore not labeled. NC: Control group; UHF‐C: IF prepared based on unhydrolyzed cow's milk WPC; UHF‐G: IF prepared based on unhydrolyzed goat's milk WPC; PHF‐C: IF prepared based on partially hydrolyzed cow's milk WPC, EHF‐C: IF prepared based on extensively hydrolyzed cow's milk WPC, PHF‐G:IF prepared based on partially hydrolyzed goat's milk WPC, and EHF‐G: IF prepared based on extensively hydrolyzed goat's milk WPC.

Based on the results obtained that butyrate was significantly reduced in allergic mice, we prioritized the correlation of butyrate content with allergic symptoms, cytokine levels, and immune cell‐related transcription factors (Figure 6b). The results showed that butyrate content was significantly negatively correlated with total IgE, specific IgE in serum, histamine, and mMCP‐1 levels (*p* < .001), suggesting that butyrate contributes to the improvement of allergic characteristics. Furthermore, butyrate content was also significantly negatively correlated with cytokines IL‐4, ‐5, and ‐17 (*p* < .001). The butyrate content was significantly and positively correlated with the mRNA expression of immune cell‐associated transcription factors T‐bet and Foxp3, while it was significantly and negatively correlated with the mRNA expression of GATA‐3 and RORγt (*p* < .001). It was implied that butyrate was regulating Th1/Th2 and Th17/Treg balance. Subsequently, we focused on the correlation between butyrate content and gut microbe and found that butyrate content was significantly positively correlated with the abundance of *Lactobacillus* (*p* < .001) and significantly negatively correlated with the abundance of *Rikenellaceae*‐RC9‐gut‐group (*p* < .001). This phenomenon is consistent with the results of microbe composition and SCFAs content in mice, where the gut of mice in the hydrolyzed IF groups was enriched with high abundance of *Lactobacillus* and low abundance of *Rikenellaceae*‐RC9‐gut‐group and produced a high content of SCFAs (butyrate).

## 4 DISCUSSION

Early life is a critical stage in the development of allergy, as the infant immune system and gut microbiota mature rapidly during this period and there is a close link between the two. External factors, including nutrition, have been reported to have the greatest impact on the long‐term immune program early in life (Cukrowska, 2018). For example, milk protein‐induced allergy in infants has received sustained attention and is a serious public health problem worldwide (Tulleken, 2018). Several therapeutic modalities, including enzymatic or lactobacilli degradation of allergens, addition of HMOs or probiotics (Tarrant & Finlay, 2023), have been used to ameliorate this problem with promising results. In this study, we focused on the most commonly used enzymatic means for industrial applications and analyzed the effect of different milk protein raw materials on allergy or post‐hydrolysis sensitization. Briefly, we found that the hydrolyzed IF significantly attenuated allergic symptoms in model mice and maintained Th1/Th2 and Th17/Treg homeostasis, which in turn modulated the expression of immune cytokines. We attribute this contribution in part to the gut microbe and its metabolic production of butyrate. In addition, we demonstrated that IF prepared from cow's milk WPC and goat's milk WPC, respectively, with the similar degree of hydrolysis (unhydrolyzed, partially hydrolyzed, or extensively hydrolyzed) did not differ in their effects on allergy in model mice. The deeper the degree of hydrolysis of cow's milk WPC or goat's milk WPC, the more obvious the effect of reducing allergic symptoms in model mice.

Milk proteins can act as allergens and induce an IgE‐mediated immune response in the host (Hussain et al., 2019). Serum levels of mMCP‐1 and histamine are often used to assess the degree of host sensitization, due to the fact that they are produced by immune cells in response to a specific outlook (e.g., allergy) (Ma, Li, et al., 2021). The antigenic epitopes of milk proteins are able to bind to IgE on host immune cells, thereby releasing mMCP‐1 and histamine to induce sensitization. This conclusion was confirmed by our results, in which high levels of mMCP‐1 and histamine were present in the serum of unhydrolyzed IF‐induced anaphylactic mice, accompanied by high levels of total IgE and specific IgE, which in turn led to the observation of significant allergic features, including high allergy scores and enlarged spleens. On the other hand, hydrolyzed IF effectively prevented these changes. Furthermore, the pathological analysis of allergy‐related tissues showed a consistent trend. Allergy induced destruction of alveolar cellular structure and infiltration of cellular inflammatory cells, and damage to jejunal villous structure, which affected the host's ability to absorb nutrients. This was ameliorated by the hydrolyzed IF. Our results are also supported by several previous studies (Noti et al., 2014; Zhao et al., 2022).

The effect of allergy on immune cells is centered on the imbalance of the T helper cell. The Th1/Th2 imbalance paradigm is fundamental and very clear. Th2 cells respond to antigens from the breast, which in turn triggers IgE production and activation of allergy‐associated immune cells, which in turn are able to promote a pro‐inflammatory cytokine cascade, producing IL‐4 and IL‐5 (Akdis et al., 2020; Caminati et al., 2018). Th1 cells, on the other hand, have been reported to produce IFN‐γ to suppress the overreaction of Th2 cells (Noti et al., 2014). This is confirmed by our results with unquestionable Th1/Th2 in allergy, although of course this imbalance is corrected when hydrolyzed IF is fed. Notably, there are also complex interactions between multiple heterogeneous T cell subsets in allergy, including Th1, Th17, T helper 22 (Th22), T follicular helper (Tfh), T helper 9 (Th9), and regulatory T cells (Tregs) (Calzada et al., 2018). In early life immune development, the presence of anergy polarizes Th2/Th17 and is accompanied by reduced Treg cell activity (Krusche et al., 2020). Further studies revealed that strengthening Treg cells inhibits allergy induced by transitionally expressed Th2 immune responses (Boonpiyathad et al., 2020). We used the expression levels of mRNA for Foxp3 and RORγt to indicate Treg/Th17 homeostasis and found that anergy caused the host to underexpress Foxp3, which in turn led to an increase in the Th17 transition represented by RORγt. The study reported a role for FoxP3 in counteracting the allergic response and an increase in the Th2 response induced by the Treg/Th17 imbalance (Wang et al., 2014). When mice were given hydrolyzed IF, we did not find extreme increases or decreases in Foxp3 and RORγt, meaning that the hydrolyzed IF helped to balance the host's Treg/Th17, which in turn blocked the allergic response. Overall, the improvement of allergic symptoms in mice by hydrolyzed IF may be achieved by regulating both Th1/Th2 and Treg/Th17 pathways together.

Early life host gut microbe is dynamic and variable and gradually matures. Gut microbe plays an important role in numerous aspects of infant health, including immune maturation, growth and development, and disease prevention (Fujimura et al., 2016). It has been found that gut microbe plays an important role in early life immune programming and its mediation of allergy development. Notably, colonization of the gut microbe early in life can influence T helper cell homeostasis (Qian et al., 2017). There may be a role for gut microbe in ameliorating milk protein allergy. Our analysis of the gut microbe in each group of mice also revealed significant differences in the composition of the gut microbe in allergic mice compared to hypoallergenic mice fed with hydrolyzed IF. First, we found a significant reduction in the abundance of *Lactobacillus* and *Alistipes* induced by allergy, whereas there was no trend toward a reduction in the hydrolyzed IF fed mice. Our observed changes in *Lactobacillus* in response to allergy are consistent with those of the study of Gong et al (2024). Undoubtedly, *Lactobacillus* has been reported to have improved gut inflammation and immune function in a variety of pathologies (Berni Canani et al., 2016; Dimattia et al., 2023). A clinical report presented that *Lactobacillus rhamnosus* GG (LGG) promoted the abundance of gut butyric acid‐producing bacteria in allergic children, which in turn resisted food allergy (Berni Canani et al., 2016). *Alistipes* is also a microorganism in the gut that has the ability to modulate host immune function and promote the production of SCFAs, suggesting that it may provide beneficial assistance to the host during allergy (Rau et al., 2018). Furthermore, we also focused on the composition of some low‐abundance microorganisms. It was found that allergy induced a significant increase in the abundance of *Rikenellaceae*‐RC9‐gut‐group, with a 5 7–5 8‐fold increase in abundance in allergic mice. Previous studies have reported that food‐allergic mice have a specific microbial profile characterized by higher abundance of *Rikenellaceae* (Gong et al., 2024; Noval Rivas et al., 2013). Unfortunately, *Roseburia*, a known class of SCFAs‐producing bacteria, presented differences between groups but did not fulfill the general pattern between allergic and non‐allergic. This is because we observed a higher abundance of *Roseburia* only in the NC and EHF‐G groups. This may be influenced by the individual specificity of the gut microbiota in mice (Mallott & Amato, 2021). In addition, we noted no significant difference in the abundance of the gut high‐abundance microorganisms *Lachnospiraceae*‐NK4A136‐group and *Muribaculaceae*, despite the fact that both accounted for half of the role of the whole microbiota. Overall, allergy alters the composition of the mouse gut microbe, promoting an increase in the abundance of the allergy‐associated *Rikenellaceae*‐RC9‐gut‐group and decreasing the abundance of the beneficial bacteria *Lactobacillus* and *Alistipes*. These beneficial microorganisms were shown to have important roles in metabolizing SCFAs and resisting host inflammation or allergy.

Short‐chain fatty acids (SCFAs) are host health beneficial metabolites produced by gut microbe metabolizing food components and have a role in preventing or ameliorating food allergies. Studies have reported that SCFAs enhance Treg cell acetylation and promote Foxp3 stability, thereby suppressing Th2 overactivated immune responses in allergy (Siller et al., 2020). In particular, butyrate, either produced by gut microbe or derived from food components (e.g., breast milk), has been reported to play a role in preventing allergy in infants (Cait et al., 2019; Paparo et al., 2021). In our results, significant differences in butyrate were observed only in groups of mice. Allergic mice were low in butyrate, whereas mice fed on hydrolyzed IF had high levels of butyrate. The change in butyrate content may explain the role of microbe in the amelioration of allergy in mice (Tyagi et al., 2018). This was demonstrated by correlation analysis of butyrate with allergic symptoms, cytokine levels, and immune cell‐associated transcription factors in mice. In addition, there was a correlation between butyrate levels and gut microbe, with *Lactobacillus* abundance positively correlating with butyrate levels. The study also reported that *Lactobacillus* metabolizes food components in the gut to produce butyrate (Tyagi et al., 2018). Moreover, butyrate content was negatively correlated with *Rikenellaceae*‐RC9‐gut‐group abundance. In a study of gut immune function, an increase in butyrate‐producing bacteria may be a key factor in reducing *Rikenellaceae*‐RC9‐gut‐group abundance (Cai et al., 2021).

Goat milk‐based formula is a better substitute for cow's milk‐based formula in infant nutrition, the lower allergenicity and better digestibility of goat's milk relative to cow's milk have been reported (Ma, Hou, et al., 2021; Zhang et al., 2022), which has been attributed to differences in α_S1_‐casein and β‐casein content in the milk (Zhang et al., 2022). Multiple studies were identified which indicate that hydrolyzed protein can reduce food allergenicity (Alexander et al., 2010; Kuo et al., 2011; Wu et al., 2014), and it is estimated that allergens must be ≥25–30 amino acids for cross‐linking of IgE receptors and activation of a pro‐inflammatory immune response (Vandenplas et al., 2019). Hydrolyzed WPC is a better‐digestible and absorbable nitrogen source than unhydrolyzed WPC, which was not digested completely by a single enzyme, and high‐molecular‐weight proteins, such as β‐lactaglobulin and α‐lactalbumin, remained (Nakano et al., 1994). In this study, we also evaluated the differences in the effects of IF prepared from cow's milk WPC and goat's milk WPC and related hydrolysates as protein ingredients on allergic effects. To the best of our knowledge, this is the first time that the effects of different milk ingredients (WPC) on allergy have been evaluated for hydrolyzed IF. The casein proteins were all from hypoallergenic goat's milk, but the whey proteins were from a different source and were brought in to assess ingredient differences. The final result we obtained was that for both cow's milk WPC and goat's milk WPC, at similar levels of hydrolysis, they did not bring about a significant effect on allergy symptoms. Hydrolyzed IF relieves allergic symptoms in mice and improves allergy‐induced Th1/Th2 and Th17/Treg imbalance. The deeper the hydrolysis degree of WPC, the better the effect of improving allergic symptoms in mice. Hydrolyzed IF was originally developed to have enhanced tolerability and reduced allergenicity, compared with intact protein formula and may therefore potentially have the benefit of decreasing the occurrence of atopic diseases (Vandenplas et al., 2019).This study provides new ideas and possibilities for the innovation of goat milk formula.

## 5 CONCLUSIONS

In conclusion, the hydrolyzed IF effectively prevented the allergic reaction in the model mice. In detail, the hydrolyzed IF reduced the serum levels of total IgE, specific IgE, histamine, and mMCP‐1, which was accompanied by low allergy scores and splenic index. We found that the hydrolyzed IF maintained Th1/ Th2 and Th17/Treg homeostasis in mice, which in turn inhibited the hydrolysis of INF‐γ and IL‐17, and promoted the levels of the anti‐inflammatory cytokines such as IL‐4, IL‐5, and IL‐10. The hydrolyzed IF also shaped the gut microbe differently from allergy, increasing *Lactobacillus* and *Alistipes* abundance while decreasing *Rikenellaceae*‐RC9‐gut‐group abundance. Further changes in the microbe modulated differences in the metabolism of SCFAs, with more butyrate in the intestines of mice fed on hydrolyzed IF. In addition, we demonstrated that for both cow's milk WPC and goat's milk WPC, at similar levels of hydrolysis, they did not bring about a significant effect on allergy symptoms. The hydrolyzed IF improved the allergic characteristics of mice, the deeper the degree of hydrolysis of WPC, the more obvious the effect of reducing allergic symptoms in model mice.

## AUTHOR CONTRIBUTIONS

**Qinggang Xie:** Conceptualization (equal); funding acquisition (equal); investigation (equal); methodology (equal); writing – review and editing (equal). **Sibo Liu:** Data curation (equal); formal analysis (equal); investigation (equal); methodology (equal); supervision (equal). **Dongying Cui:** Investigation (equal); methodology (equal); writing – original draft (equal). **Yang Liu:** Investigation (equal); methodology (equal); writing – original draft (equal). **Xiangxin Wang:** Investigation (equal); methodology (equal); writing – original draft (equal). **Ting Cao:** Investigation (equal); methodology (equal); writing – original draft (equal). **Xiaoxi Xu:** Conceptualization (equal); funding acquisition (equal); project administration (equal); resources (equal); writing – review and editing (equal). **Bailiang Li:** Conceptualization (equal); methodology (equal); project administration (equal); supervision (equal); writing – review and editing (equal).

## CONFLICT OF INTEREST STATEMENT

The authors declare that they have no known competing financial interests or personal relationships that could have influenced the work reported in this study.

## Data Availability

Data will be made available on request.
